# Lymphangioma‐Induced Macroglossia in an Adolescent: A Case Report

**DOI:** 10.1002/ccr3.72249

**Published:** 2026-03-12

**Authors:** Arezoo Heidary, Mojtaba Mehrabanian

**Affiliations:** ^1^ Department of Oral and Maxillofacial Diseases, Faculty of Dentistry Yazd Shahid Sadoughi University of Medical Sciences Yazd Iran; ^2^ Dental Research Center, Dental Research Institute, School of Dentistry Isfahan University of Medical Sciences Isfahan Iran

**Keywords:** hamartoma, lymphangioma, lymphatic malformation, macroglossia, sclerotherapy

## Abstract

Lymphangioma is a congenital malformation of the lymphatic system, commonly affecting the head and neck region. Macroglossia due to lymphangioma in adolescence is uncommon, making this case noteworthy. This condition can affect oral function, speech, and quality of life, making early diagnosis and intervention essential. This report highlights a 14‐year‐old Balouch female presenting with progressive tongue enlargement beginning at age five. Intraoral examination revealed an enlarged tongue with a pebbly surface, particularly involving the ventral side of the tongue, without osseous involvement on radiographs. Differential diagnoses included hemangioma and lymphangiomegaly; however, histopathological analysis confirmed lymphangioma. The patient underwent sclerotherapy, resulting in a significant reduction in tongue size, improved oral function, and resolution of related symptoms. Follow‐up revealed no recurrence or major complications. Early recognition and appropriate management of lymphangioma‐induced macroglossia are essential to prevent functional limitations and improve quality of life. While surgical excision remains the conventional treatment, sclerotherapy offers a less invasive, effective alternative with lower morbidity. This case contributes to the limited literature on adolescent‐onset oral lymphangioma and underscores the value of a multidisciplinary approach in ensuring optimal outcomes.

## Key Clinical Message

Adolescent‐onset macroglossia caused by lymphangioma is rare but treatable. Early diagnosis and minimally invasive sclerotherapy can successfully restore function, reduce morbidity, and prevent recurrence. It can be a viable alternative to surgery in carefully selected cases.

## 1 Introduction

Lymphangioma is a congenital malformation of the lymphatic system that most frequently affects the head and neck region, but it may also appear in other parts of the body. In some cases, it can expand into surrounding anatomical structures and cause life‐threatening complications [1]. Lymphangiomas are typically present at birth or appear in early childhood [2]. It has been reported that approximately 75% of all lymphangiomas occur in the head and neck region. Notably, about 50% of cases are evident at birth, and approximately 90% are diagnosed by the age of 2 years [3, 4, 5]. Diagnosis beyond early childhood is uncommon, making adult‐onset lymphangioma a rare clinical finding [4].

In the oral cavity, lymphangiomas are most commonly found in the anterior two‐thirds of the tongue [4], where they may appear red or purple in color. However, they have also been reported on the palate, gingiva, buccal mucosa, lips, and mandibular alveolar ridge [4]. Oral lymphangiomas often present as translucent plaques composed of small, thin‐walled vesicles that resemble frog eggs. These lesions typically display a pebbly or irregularly nodular surface and vary in color from gray to pink or yellowish, depending on their depth and location [5].

Clinically, superficial lymphangiomas are characterized by their translucent hue and irregular surface texture, while deeper lesions tend to present as soft, diffuse nodular masses that may appear red or blue, possibly due to the rupture of blood capillaries into the lymphatic space [5]. They may appear as localized or diffuse growths. Larger lesions can cause macroglossia, impaired speech, and difficulty in mastication and swallowing [6]. A pathognomonic feature of tongue lymphangioma is macroglossia [5]. Lymphangiomas are classified based on vessel size into capillary, cavernous, and cystic types [4].

Although history and physical examination are often sufficient for diagnosis, lymphangiomas may require differentiation from other lesions. The differential diagnosis includes hemangioma, dermoid cyst, teratoma, amyloidosis, thyroglossal duct cyst, neurofibromatosis, granuloma, congenital hypothyroidism, primary muscular hypertrophy, lipoma, neurofibroma, salivary and thyroid gland tumors, heterotopic gastric mucosal cyst, and meningoencephalocele [5].

Surgical resection is the most effective treatment and generally has a good prognosis. However, complications such as airway obstruction and even death may occur depending on the lesion size and location [5]. Although surgical resection is the treatment of choice, complications such as damage to surrounding structures, including nerves and blood vessels, scarring, and recurrence due to incomplete removal can be significant. Intralesional injection of sclerosing agents, such as bleomycin, tetracycline, alcohol, and OK‐432, has been suggested as an alternative treatment [1].

This case highlights a unique presentation of tongue lymphangioma leading to macroglossia in a 14‐year‐old Balouch girl, contributing to existing literature by documenting a rare late‐onset manifestation.

## 2 Case History/Examination

A 14‐year‐old Balouch female patient was referred to the Department of Oral and Maxillofacial Medicine at Shahid Sadoughi University of Medical Sciences, Yazd, Iran, with a chief complaint of tongue enlargement. According to her parents, the tongue had begun to increase in size around the age of five. They noted occasional bleeding, which was attributed to repeated minor mechanical trauma, including inadvertent tongue biting during mastication and chronic friction between the enlarged tongue and adjacent dentition, leading to rupture of fragile superficial lymphatic vesicles. The recent worsening of the swelling had also caused breathing difficulties.

Her medical history showed no ongoing systemic conditions. However, she had a history of two unexplained childhood seizures and had been treated with corticosteroids for 1 year due to bulging leg veins. There were no significant genetic conditions reported in the family history, and no prior medical interventions before the referral.

**FIGURE 1:**
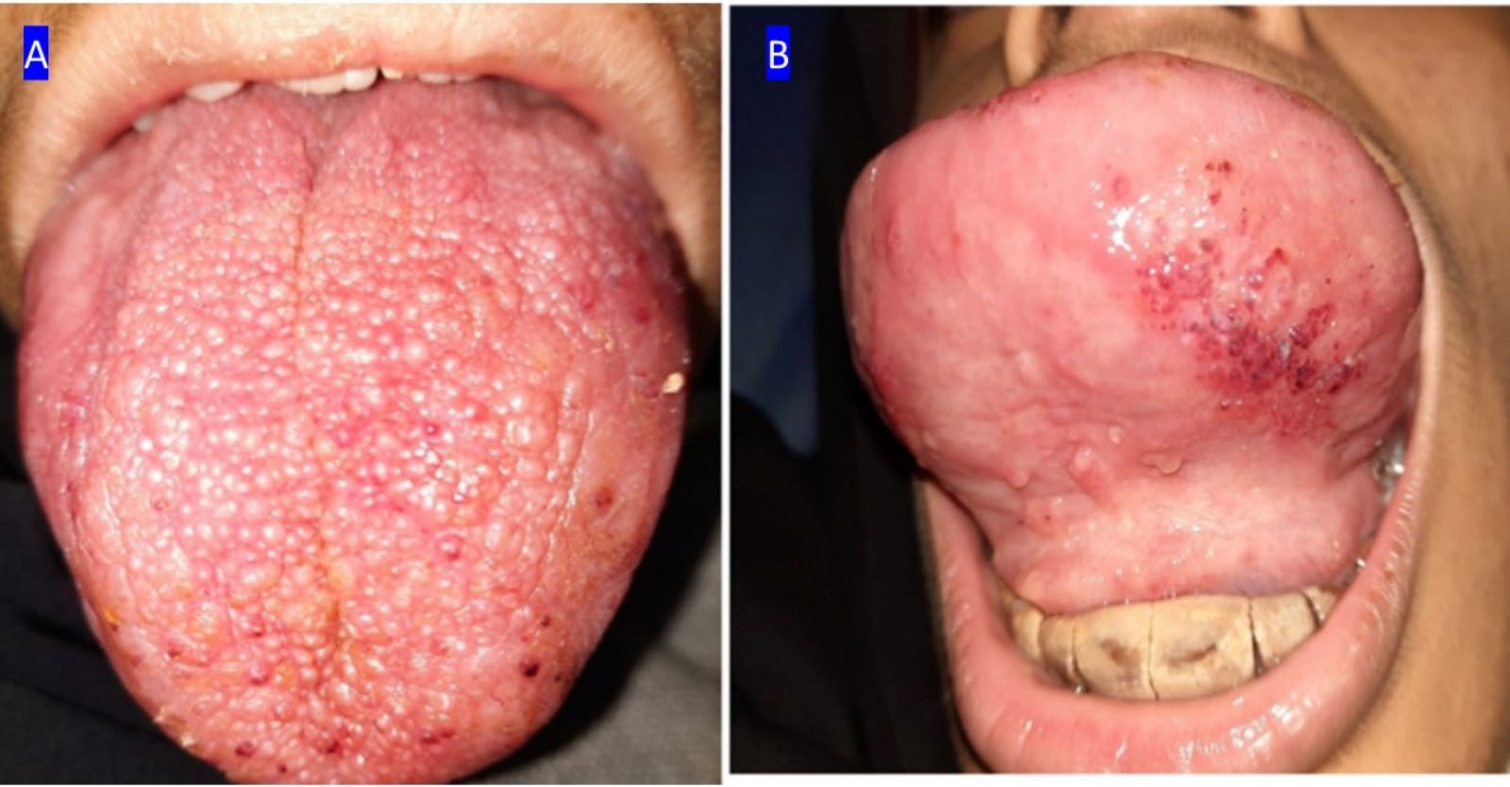
Intraoral views of the tongue. (A) Dorsal view showing diffuse tongue enlargement with a pebbly, nodular surface. (B) Ventral view displaying pronounced tongue protrusion and surface irregularities, with focal areas of hemorrhagic discoloration due to trauma.

Intraoral examination revealed a markedly enlarged tongue with a pebbly surface, involving the ventral side of the tongue. Consequences included tooth dislocation, open bite, and heavy calculus accumulation (Figure 1). Café‐au‐lait spots were observed on the body.

## 3 Methods

**FIGURE 2:**
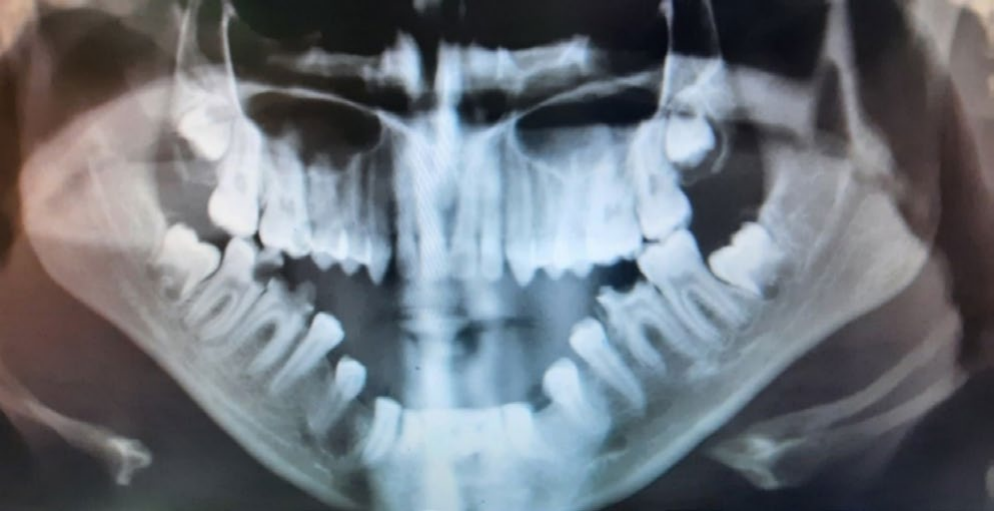
Panoramic radiograph showing no osseous involvement.

**FIGURE 3:**
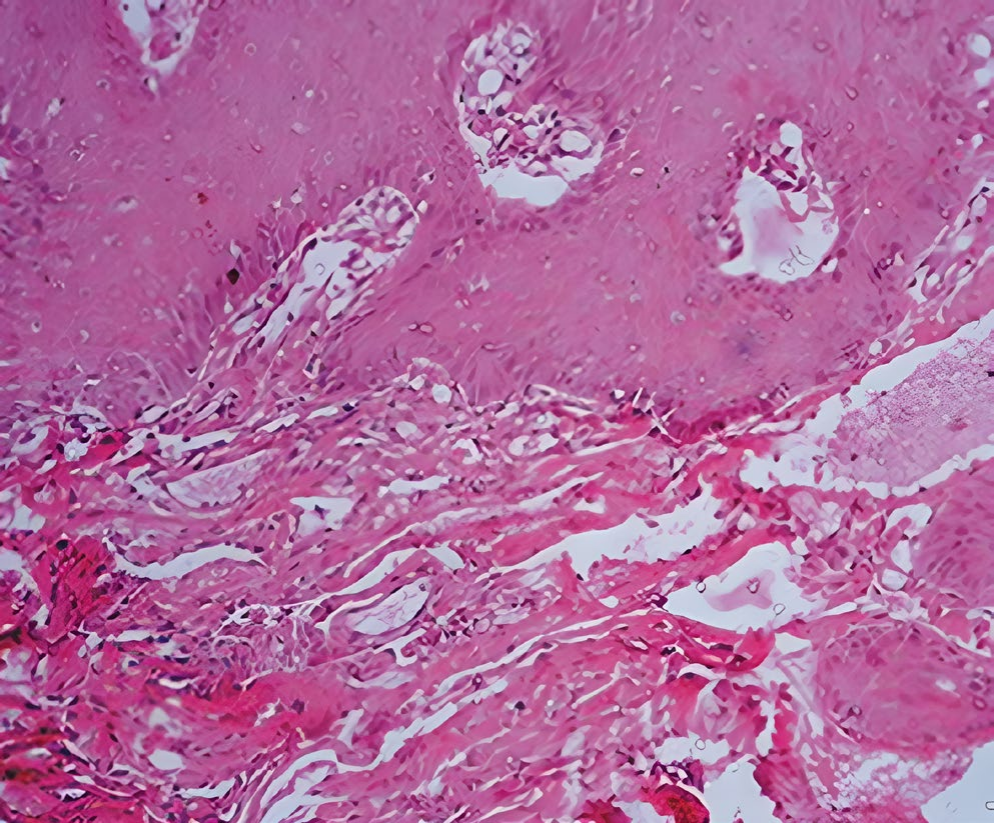
Histopathologic image showing dilated lymphatic vessels.

For a better assessment, a panoramic radiograph was performed, which demonstrated no evidence of osseous involvement (Figure 2). The patient was referred to a maxillofacial surgeon with a differential diagnosis of hemangioma and lymphangiomegaly. An incisional biopsy was performed. Microscopic examination of the submitted sample revealed muscle and connective tissues interspersed with numerous small and large dilated, cystic lymphatic vessels lined by endothelium. Lymphatic spaces were also visible beneath the surface epithelium (Figure 3). Based on the histopathological findings, a definitive diagnosis of lymphangioma was established.

## 4 Results

Following the histopathological confirmation of lymphangioma, the patient underwent intralesional sclerotherapy performed by a maxillofacial surgeon. Bleomycin was used as the sclerosant, diluted with normal saline to a concentration of 1 mg/mL and administered intralesionally at a dose of 0.5 mg/kg, with the cumulative dose maintained below 5 units/kg. Treatment sessions were performed at 6‐week intervals, for a total of four sessions. This dosing regimen and treatment interval are consistent with previously reported pediatric head and neck lymphangioma protocols using intralesional bleomycin [7].

Progressive clinical improvement was observed, with satisfactory resolution achieved within approximately 12 months. Clinically, the patient demonstrated significant improvement in oral function, including speech articulation, mastication, and swallowing. The reduction in tongue size alleviated previously reported breathing difficulties and minimized trauma‐related bleeding episodes.

The tongue showed reduced protrusion and less surface irregularities compared to pretreatment photographs (Figure 1). There were no signs of lesion recurrence, infection, or other complications. The patient and her family reported satisfaction with both the functional and aesthetic outcomes.

Table 1 summarizes the patient's clinical timeline from symptom onset to diagnosis, treatment, and 1‐year follow‐up outcome.

**TABLE 1:** Timeline of the patient's symptom progression, diagnosis, treatment and follow‐up for tongue lymphangioma.

Age	Clinical event
5 years	Initial tongue swelling noticed by parents
10 years	Increased tongue size with occasional bleeding episodes
13 years	Symptoms worsened, difficulty in speaking and chewing
14 years	Referred to a maxillofacial surgeon. Histopathology confirmed lymphangioma
15 years	1‐year post‐treatment follow‐up: Marked reduction in tongue size, improved oral function, no recurrence

## 5 Discussion

Lymphangioma is a benign hamartomatous growth. Typically, it is a rare condition and is most often diagnosed during infancy [8]. It is most commonly found in the head and neck, while oral manifestations are rare [9]. Lymphangiomas of the tongue are relatively uncommon [10]. Clinically, these lesions present as painless, slow‐growing soft swellings [11].

Although most cases begin in infancy or early childhood (before the age of two), some studies document cases in older individuals, often with a male predilection. Vasconcelos et al. reported a 17‐year‐old female with persistent oral blisters for 1 year, associated with bleeding during feeding [4]. Kolay et al. described three lymphangioma cases, including a 6‐year‐old boy, a 13‐day‐old infant, and a 2‐year‐old with tongue involvement and functional impairment [5]. Hasan et al. reported a 45‐year‐old male with a gradually enlarging lesion over 3 years [12].

Other reported cases include a 23‐year‐old male with buccal mucosa swelling [13], a 5‐year‐old girl with lateral tongue growth and masticatory trauma [14], and a 4‐year‐old with macroglossia and airway obstruction [15].

Tongue lymphangiomas are most commonly observed in the anterior portion of the tongue [4]. Partial involvement of the tongue has been reported by Kolay et al., Goswami et al., and Farronato et al. [5, 14, 15]. However, in our case, the lesion involved the entire tongue, which is an uncommon presentation.

Furthermore, our findings of a pebbly surface on the ventral tongue align with previous reports by Bhayya et al. and Nelson et al. [6, 16].

This report presents a rare case of adolescent‐onset tongue lymphangioma confirmed by histopathology and successfully managed with minimally invasive sclerotherapy. The multidisciplinary approach and detailed clinical timeline are notable strengths. However, the absence of advanced imaging, objective functional assessments, and long‐term follow‐up limits the generalizability and depth of outcome evaluation. Future studies involving larger cohorts and standardized outcome measures are needed to support broader clinical application.

## 6 Conclusions

Early diagnosis and appropriate management of lymphangioma‐induced macroglossia in adolescents can prevent complications. While surgical resection is the standard treatment, minimally invasive options like sclerotherapy provide effective alternatives with lower risks. A multidisciplinary approach is key to optimizing outcomes.

## Author Contributions

**Arezoo Heidary:** conceptualization, data curation, investigation, methodology, writing – review and editing. **Mojtaba Mehrabanian:** writing – original draft, writing – review and editing.

## Funding

The authors have nothing to report.

## Ethics Statement

Ethical approval for this study was obtained from the Shahid Sadoughi University of Medical Sciences, Yazd, with the ethics code IR.SSU.DENTISTRY.REC.1403.103. Written informed consent was obtained from the patient's guardian.

## Consent

Written informed consent was obtained from the patient for the publication of this case report and any accompanying images. A copy of the written consent is available for review by the Editor‐in‐Chief of this journal.

## Conflicts of Interest

The authors declare no conflicts of interest.

## Data Availability

All data supporting the findings of this case report are included within the manuscript. Further details are available from the corresponding author upon reasonable request.
